# Six and eight weeks injection frequencies of bevacizumab are non-inferior to the current four weeks injection frequency for quality of life in neovascular age-related macular degeneration: a randomized controlled trial

**DOI:** 10.1007/s11136-020-02580-9

**Published:** 2020-07-14

**Authors:** Martijn S. Visser, Sankha Amarakoon, Tom Missotten, Reinier Timman, Jan J. V. Busschbach

**Affiliations:** 1 Department of Psychiatry, Section Medical Psychology and Psychotherapy, Erasmus Medical Center, PO Box 2040, 3000 CA Rotterdam, The Netherlands; 2 Rotterdam Ophthalmic Institute, Rotterdam, The Netherlands

**Keywords:** Age-related macular degeneration, Bevacizumab, Injection frequency, NEI VFQ-39, Quality of life

## Abstract

**Purpose:**

Patients with neovascular age-related macular degeneration (nARMD) will not deteriorate on visual acuity and retinal thickness when treated with bevacizumab injection frequencies of 6 or 8 weeks compared to 4 weeks. This study aimed to investigate this non-inferiority in quality of life (QoL). We hypothesized that less frequent bevacizumab injections are not inferior regarding patients reported QoL.

**Methods:**

Patients were randomized to bevacizumab every 4 (*n* = 64), 6 (*n* = 63), and 8 weeks (*n* = 64). Patients were at least 65 years old, have a best-corrected visual acuity of 20/200 to 20/20, no previous ARMD treatment and active leakage. Vision-related QoL questionnaire NEI VFQ-39 was used to assess QoL at baseline and after 1 year. General QoL questionnaire SF-36 was included for secondary analysis. Multilevel analyses were performed, correcting for age, gender and baseline.

**Results:**

The 6 (3.68; 95% CI − 0.63 to 8.00) and 8 (2.15; 95% CI − 2.26 to 6.56) weeks bevacizumab regimens resulted in non-inferior QoL differences compared to 4 weeks on the NEI VFQ-39. Also on the SF-36 the differences were well within the non-inferiority limits.

**Conclusion:**

Non-inferiority of the 6 and 8 weeks frequencies was demonstrated compared to 4 weeks on vision-related and general QoL in patients with nARMD. These results are in line with previously published results of lower frequency injections regarding visual acuity and central retinal thickness. Lower injection frequency may reduce burden, side effects, and treatment costs. In consideration of these results, 8 weeks frequency injections of intravitreal bevacizumab could be considered in patients with nARMD.

**Electronic supplementary material:**

The online version of this article (10.1007/s11136-020-02580-9) contains supplementary material, which is available to authorized users.

## Introduction

Age-related macular degeneration (ARMD) is the leading cause of severe vision loss and blindness among people aged over 50 years in Western countries [1, 2]. ARMD affects central retinal function, profoundly impairing the patient’s ability to perform daily activities and their quality of life (QoL) [3]. Exudative ARMD, an aggressive form of ARMD [4, 5], progresses rapidly and is characterized by the development of choroidal neovascularization (CNV); hence, it is often described as neovascular ARMD (nARMD). The current standard therapy for nARMD is intravitreal injection of anti-vascular endothelial growth factor (VEGF), a treatment which improves the visual prognosis of nARMD patients considerably.

To enhance effective patient-centered care, there is a trend toward gathering outcome information from the patient’s perspective in addition to the clinical outcomes. Since there is interest in the patients’ perspective of satisfaction, in terms of outcome, several patient-reported outcome measures (PROMs) have been developed [6]. Several studies have suggested that the use of PROMs have a positive effect on the doctor-patient communication, and consequently patients’ satisfaction [7].

The most commonly used anti-VEGF medications are ranibizumab, aflibercept and bevacizumab. The efficacy of ranibizumab and aflibercept has been proven and appear clinically equivalent, and are approved both by the Food and Drug Administration (FDA) and the European Medicines Agency (EMA) for intraocular use in nARMD [8–13]. Bevacizumab has been approved by the FDA and the EMA for the treatment of various tumors, such as colorectal cancer [14], but not specifically for nARMD. However, in recent years, ophthalmologists have been prescribing bevacizumab for off-label use in nARMD because it is a cost-effective substitute for ranibizumab and aflibercept [15–20]. Multiple studies provided RCT evidence supporting the efficacy of bevacizumab in a monthly, pro re nata and treat-and-extend regimes [15–20]. The CATT study also showed that there is no difference in effectiveness in term of vision and side effects between ranibizumab and bevacizumab and is comparably effective when the injection frequency is 4 weeks. Moreover, the IVAN study showed similar results on QoL for bevacizumab and ranibizumab measured with the EuroQol-5D [21], macular disease-specific quality of life [22] and treatment satisfaction [23].

The every-four-weeks regimen used in the CATT study was chosen for bevacizumab based on prior ranibizumab trials and is a widely adopted and proven strategy. However, the relatively long half-life of bevacizumab might allow the achievement of a therapeutic effect with less frequent injections, as has been the experience in the clinic [24, 25]. Reduced numbers of injections could have several beneficial effects, including a decrease in the risks associated with intravitreal injection (such as endophthalmitis and retinal detachment), improved cost-effectiveness, reduced patient burden, and a reduced ophthalmic work-load. A study in nARMD patients comparing an every-four-weeks injection frequency of bevacizumab therapy to an every-six-weeks or every-eight-weeks injection frequency showed no significant difference for lower injection frequencies for visual acuity and central retinal thickness [26]. In the current non-inferiority study, we aimed to determine whether bevacizumab therapy administered every 6 or 8 weeks is also not inferior to an every-four-weeks regimen for QoL outcomes in nARMD patients.

## Materials and methods

### Study patients

**Fig. 1:**
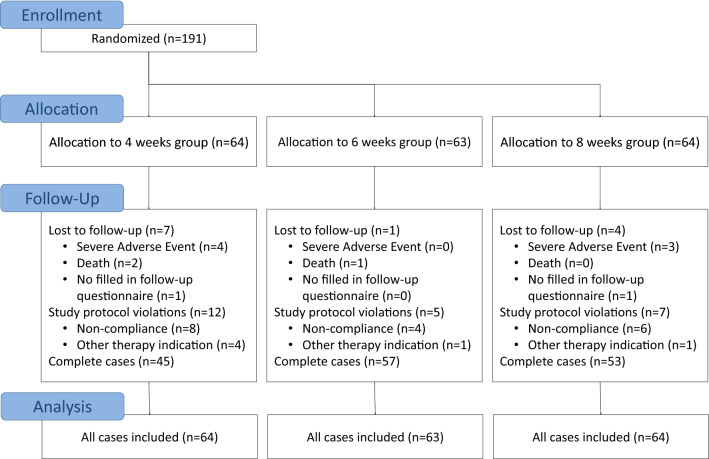
CONSORT flow diagram of enrolment, allocation, follow-up and analysis of the every-four-weeks, every-six-weeks, and every-eight-weeks treatment groups [38]

This is a secondary analysis of an RCT comparing three treatment regimens of bevacizumab (Avastin) for the treatment of ARMD on visual acuity and central retinal thickness [26]. A total of 191 patients were enrolled in a 1-year, prospective, open-label RCT which investigated the optimal injection frequency of bevacizumab injection for ARMD treatment at the Rotterdam Eye Hospital from June 2008 to March 2010 (Fig. 1). To be eligible, patients had to be at least 65 years old, have a best-corrected visual acuity of 20/200 to 20/20 (Snellen equivalent) in the study eye as assessed using Early Treatment Diabetic Retinopathy Charts (ETDRS), no previous ARMD treatment and active leakage. Patients were only treated in one eye. Fluorescein angiography (FA) and indocyanine green (ICG) angiography were used to observe leakage, and optical coherence tomography (OCT) was used to observe the presence of fluid [26]. Patients who had other significant ocular disorders, had allergies to either FA or ICG dye injections, were immunocompromised, using coumarin-derivatives, had experienced a clinically significant cerebrovascular accident or myocardial infarction or had a planned ocular surgery during the 1-year follow-up, were excluded. Written informed consent was obtained from all participants. After baseline measurements were completed, all eligible patients were randomized to an injection frequency of every 4, 6, or 8 weeks using a computer-based 1:1:1 ratio block randomization procedure.

### Treatment

Apart from the difference in frequency, treatment regimens were comparable among the three groups. At each outpatient visit, a dose of 1.25 mg bevacizumab was administered intravitreally. On top of the measures during regular outpatient visits, patients were assessed every 12 weeks by best-corrected visual acuity, spectral-domain OCT and funduscopy. Monthly checks for adverse events took place by questioning patients. Treatment was continuous for 1 year, independent of visual acuity change, spectral-domain OCT measures, or funduscopy findings. The 4 weeks, 6 weeks, and 8 weeks bevacizumab treatment regimens resulted in totals of 13, 9, and 7 injections and visits a year, respectively.

### Outcome measures

At baseline and at the final follow-up visit, patients were asked to complete the National Eye Institute 39-Item Visual Function Questionnaire (NEI VFQ-39) [27] and the 36-item Medical Outcomes Study Short-Form General Health Survey (SF-36) [28, 29]. The NEI VFQ-39 assesses vision-related QoL, while the SF-36 evaluates general QoL. Given the nature of the disease, both questionnaires were presented in a larger font size and often administered in the presence and sometimes with support of a caregiver and/or family member.

### Vision-related quality of life: NEI VFQ-39

The primary outcome was vision-related QoL, measured as the composite score on the NEI VFQ-39 [27]. The NEI VFQ-39 consists of a 25-item base set of questions and 14 supplemental items. All items use a Likert-type scaling and five response categories, with occasionally a sixth category to opt out, except for two items that have 10 response options. Responses are converted into 12 vision-targeted multi-item subscales (0–100): general health, general vision, ocular pain, near activities, distant activities, social functioning, mental health, role limitations, dependency, driving, color vision, and peripheral vision. These 12 subscales can be summarized as a single composite score. A 10-point difference in either the sub-scales or the composite score of the NEI VFQ-39 is deemed clinically important, and thus considered a clinically meaningful change [30, 31]. The reliability of the NEI VFQ-39 in age-related macular degeneration varies from a Cronbach’s alpha of 0.86 to 0.96 [32, 33].

### General quality of life: SF-36

Another outcome measure was general QoL measured by the SF-36 [29]. This is a self-report questionnaire comprising 36 questions measuring different aspects of general health. All items use a Likert based scaling and use two to six response options. The responses are converted into eight multi-item subscales: physical functioning, role functioning physical, bodily pain, general health, vitality, social functioning, role functioning emotional, and mental health. These scales can be summarized as a psychometrically based ‘physical component summary’ (PCS), in which the first four scales are most heavily weighted, and a ‘mental component summary’ (MCS), in which the last four scales are most heavily weighted [34]. These summaries are transformed into T-scores with a mean of 50 and standard deviation of 10. Higher scores on SF-36 scales indicate a better quality of life. The UK version reliability of the physical subscale is 0.92, and the mental subscale is 0.89 [34]. Following the approach provided by Jacobson & Truax, the clinical significant change is 7.84 and 9.19 for the respective subscales [35].

### Data analysis and statistical methods

**Fig. 2:**
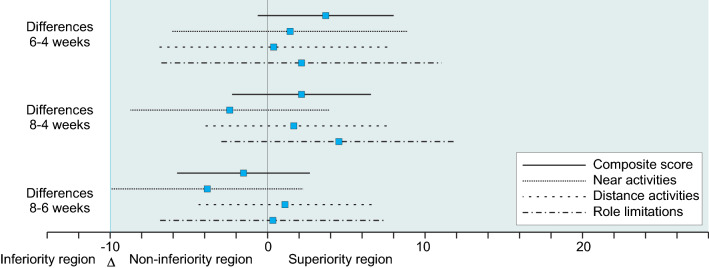
Forest plot of 95% confidence intervals of differences between treatment groups. The sensitivity analysis was based on a matched sample

Differences between dropouts and retained patients were analyzed with Student’s *t*- and chi square-tests. Baseline differences for continuous variables between the three groups were analyzed with One-way ANOVA with Bonferroni correction for pairwise differences. Chi square-tests were applied for binary variables and when significant, standardized residuals were evaluated to determine the deviating groups. The non-inferiority limit for the 6 weeks and 8 weeks groups comparison with the 4 weeks group was based on the 10-point clinical significant difference of the NEI VFQ, composite score and the subscales near vision, distance vision and role limitations. This negative 10-point difference indicated the lower end of the ‘region of therapeutic equivalence’ and, together with the maximum possible difference, enclosed the ‘region of non-inferiority’ [36]. The region of non-inferiority ranged from − 10 to 100. Non-inferiority was assumed whenever the 95% confidence interval of the difference in change fell entirely within this region [36]. Note that only the right-hand side of the distribution was relevant, Fig. 2.

In addition, differences between treatment groups were tested for the secondary SF-36 subscales. We applied multilevel linear regression analyses to evaluate differences in change in QoL between the three randomization groups. The patients formed the upper level, their repeated measures the lower level. These analyses can handle data with missing time points efficiently, i.e. data of patients without a follow-up can be included, without a need for imputation. For each outcome we applied a separate model. The random parts of the models only included the intercept. The fixed parts of the models included time (follow-up vs. baseline), centered baseline score, 6-weeks and 8-week frequencies and the interaction of time with baseline, six and eight weeks frequencies. The four-week frequency group served as reference group. In all analyses, gender and age were included as control variables.

The study was originally designed to detect differences in visual acuity, and subsequently powered with a non-inferiority limit of seven letters [26]. When testing QOL, a power analysis for non-inferiority was performed on the NEI VFQ-39 composite score. The clinical important difference for the NEI VFQ-39 is 10 and the standard deviation is 20, the one-sided alpha was set at 0.05 and power at 0.80, for which a sample size of 50 persons per group is needed. This implies that the sample size of 63–64 is sufficient.

All other analyses were performed with IBM SPSS version 24.0 “IBM Corp. Released 2016. IBM SPSS Statistics for Windows, Version 21.0. Armonk, NY: IBM Corp.”

This study was approved by the Erasmus Medical Research Ethics Committee (MEC-2007-254) in accordance with the Code of Ethics of the World Medical Association (Declaration of Helsinki) and was registered in the Dutch Trial Register (NTR 1174).

## Results

### Demographic and clinical characteristics

After randomization, 64 patients were treated in the 4 weeks group, 63 in the 6 weeks group, and 64 in the 8 weeks group. Treatment arms were well balanced with regard to baseline demographic characteristics, visual acuity, and other characteristics of the affected eye (Table 1). However, significant baseline differences were present for the NEI VFQ-39 as the 8 weeks group had lower scores than the 4 weeks group.

**Table 1:** Baseline characteristics

Characteristics	Every 4 weeks (*n* = 64)	Every 6 weeks (*n* = 63)	Every 8 weeks (*n* = 64)	*p* value
Age in years at baseline, mean ± SD	76.5 ± 6.8	77.4 ± 6.7	78.1 ± 6.1	0.436
Gender, male *n* (%)	18 (28.1)	25 (39.7)	21 (32.8)	0.382
Race, Caucasian *n* (%)	63 (98.4)	63 (100)	64 (100)	0.369
Visual acuity score ± no. letters	66 ± 12	65 ± 13	62 ± 15	0.230
Total thickness at fovea, µm ± SD	369 ± 85	371 ± 97	371 ± 97	0.990
Patients treated in worse eye, *n* (%)^a^	30 (56.6)	31 (56.4)	30 (53.6)	0.955
NEI VFQ-39, mean ± SD^b,e^				
Composite score	72.0 ± 17.6	67.8 ± 20.0	63.1 ± 19.4	0.032^f^
Near activities	60.5 ± 24.2	57.1 ± 24.7	49.4 ± 26.7	0.041^f^
Distant activities	67.8 ± 23.6	64.3 ± 25.2	57.9 ± 25.1	0.073
Role limitations	64.2 ± 25.9	60.2 ± 27.2	52.6 ± 25.5	0.042^f^
SF-36, mean ± SD^b,c,e^				
Physical component	44.8 ± 10.9	42.1 ± 11.1	42.2 ± 9.2	0.288
Mental component	50.9 ± 9.1	51.5 ± 11.5	48.4 ± 11.1	0.239
Lost to follow-up, *n* (%)				
Exit reason				
SAE^d^	4 (6.3)	0 (0.0)	3 (4.7)	0.150
Death	2 (3.1)	1 (1.6)	0 (0.0)	0.364
Non-compliance	8 (12.5)	4 (6.3)	6 (9.4)	0.495
No filled in follow-up questionnaire	1 (1.6)	0 (0.0)	1 (1.6)	0.608
Other therapy indication	4 (6.3)	1 (1.6)	1 (1.6)	0.217
Total	19 (29.7)	6 (9.5)	11 (17.2)	0.013^g^

^a^The treatment eye was defined as the worse-seeing eye when the visual acuity letter score at baseline was worse by five or more letters compared to that for the fellow eye. Patients with missing visual acuity (VA) scores or similar VA scores within a 5 letter range, were omitted, resulting in *n* = 53, 55, and 56, respectively [38]

^b^Higher scores indicate a better quality of life

^c^In the every 4 weeks group *n* = 63

^d^SAE = Severe Adverse Event

^e^The baseline scores were included in the multilevel model to adjust for potential differences

^f^The difference was between the every 4–8 weeks *p* < 0.05 (Bonferroni correction)

^g^Every 4 weeks is overrepresented

### Dropouts

Patients lost to follow-up were subdivided based on their exit reasons (Table 1). The highest drop-out rate in the 4 weeks treatment group (29.7%) and the lowest in the 6 weeks group (9.5%) significantly differed, *p* = 0.004. Patients who dropped out had significantly worse baseline scores than retained patients on the physical component summary of the SF-36: *t*(176) =  − 2.95, *p* = 0.004 (not in Table 1). No other statistical significant differences were found.

### NEI VFQ-39

The changes and differences estimated by the multilevel models are presented in Table 2, the total models are presented in Table 3. Observed differences are presented in Appendix 1 and the observed means and standard deviations in Appendix 2. The 95% confidence intervals of the difference in change scores showed that the composite score interval was well inside the [− 10, 100] point difference interval that represented the non-inferiority region for the three treatment comparisons (Fig. 2). For the subscales near activities, distant activities, role limitations, visual functioning and socio-emotional functioning the 95% confidence intervals of the differences were also entirely within the region of non-inferiority. This also barely holds for the near activities estimate for gain within 6 weeks (10.26) compared to gain within 8 weeks (6.43). This 95% confidence interval of − 9.91 to 2.24 is just within the limit.

**Table 2:** Estimated changes in the NEI VFQ-39 and SF-36 scores, age, gender and baseline controlled score

	Every 4 weeks	Every 6 weeks	Every 8 weeks	Differences 6–4 weeks		Differences 8–4 weeks		Differences 8–6 weeks	
	Estimate [95% CI]	Estimate [95% CI]	Estimate [95% CI]	Estimate [95% CI]	*p* value	Estimate [95% CI]	*p* value	Estimate [95% CI]	*p* value
NEI VFQ-39									
Composite score	2.91	6.59	5.05	3.68	0.094	2.15	0.338	− 1.53	0.474
	[− 0.25, 6.07]	[3.65, 9.53]	[2.04, 8.07]	[− 0.63, 8.00]		[− 2.26, 6.56]		[− 5.75, 2.68]	
Near activities	8.83	10.26	6.43	1.43	0.650	− 2.41	0.452	− 3.84	0.215
	[4.33, 13.34]	[6.01, 14.51]	[2.10, 10.75]	[− 4.76, 7.62]		[− 8.71, 3.89]		[− 9.91, 2.24]	
Distance activities	5.13	5.51	6.80	0.38	0.898	1.66	0.580	1.28	0.656
	[0.90, 9.37]	[1.56, 9.47]	[2.74, 10.85]	[− 5.41, 6.17]		[− 4.24, 7.56]		[− 4.39, 6.60]	
Role limitations	1.63	5.83	6.15	4.21	0.259	4.53	0.234	0.32	0.929
	[− 3.73, 6.98]	[0.84, 10.82]	[1.04, 11.27]	[3.11, 11.52]		[− 2.94, 11.99]		[− 6.83, 7.48]	
SF-36									
Physical component	− 0.88	− 0.53	− 0.25	0.35	0.810	0.63	0.670	0.28	0.842
	[− 3.00, 1.23]	[− 2.49, 1.42]	[− 2.26, 1.76]	[− 2.52, 3.22]		[− 2.29, 3.56]		[− 2.52, 3.09]	
Mental component	3.20	0.85	2.66	− 2.35	0.103	− 0.54	0.715	1.82	0.201
	[1.13, 5.27]	[− 1.09, 2.78]	[0.66, 4.66]	[− 5.18, 0.47]		[− 3.42, 2.35]		[− 0.97, 4.60]	

Non-inferiority means that the lower boundary of the 95% CI is not lower than minus 10 for the NEI-VFQ composite score or subscales and *p* values indicate whether the difference of changes is different from zero

**Table 3:** Multilevel VFQ-39 and SF-36 models

	NEI VFQ-39								SF-36			
	Composite score		Near activities		Distance activities		Role limitations		Physical component		Mental component	
	Estimate	*p* value	Estimate	*p* value	Estimate	*p* value	Estimate	*p* value	Estimate	*p* value	Estimate	*p* value
Intercept	67.02	< 0.001	54.70	< 0.001	62.37	< 0.001	58.02	< 0.001	42.94	< 0.001	50.13	< 0.001
Male	2.21	0.024	3.40	0.014	3.36	0.012	3.49	0.030	0.56	0.383	0.21	0.739
Age	0.00	0.991	− 0.09	0.371	− 0.07	0.511	0.00	0.984	0.05	0.303	− 0.08	0.080
Time	2.91	0.071	8.83	< 0.001	5.13	0.018	1.63	0.551	− 0.88	0.412	3.20	0.003
Baseline	1.00	< 0.001	0.99	< 0.001	0.99	< 0.001	0.99	< 0.001	1.00	< 0.001	1.00	< 0.001
Time × baseline	− 0.32	< 0.001	− 0.29	< 0.001	− 0.31	< 0.001	− 0.38	< 0.001	− 0.28	< 0.001	− 0.54	< 0.001
Every 6 weeks	− 0.21	0.885	− 0.25	0.907	− 0.21	0.917	− 0.37	0.881	− 0.14	0.887	0.09	0.920
Time × 6 weeks	3.68	0.094	1.43	0.650	0.38	0.898	4.21	0.259	0.35	0.810	− 2.35	0.103
Every 8 weeks	− 0.13	0.930	− 0.14	0.946	− 0.20	0.920	− 0.18	0.942	− 0.12	0.900	0.16	0.869
Time × 8 weeks	2.15	0.338	− 2.41	0.452	1.66	0.580	4.53	0.234	0.63	0.670	− 0.54	0.715

### SF-36

The treatment did not significantly affect the SF-36 component summaries. All treatment effects of different injection frequencies were well within the non-inferiority limits (Table 2).

## Discussion

To study non-inferiority of a less frequent injection schedule for bevacizumab therapy, we tested QoL in 191 ARMD patients who were randomly assigned to receive 1 year of continuous treatment with intravitreal bevacizumab injections every 4, 6, or 8 weeks. In this study we showed that 6 weeks and 8 weeks injection regimens were not inferior to the four-week regimen in QoL assessments. The eight-week regimen was also not inferior to the six-week regimen. Thus, regarding patient satisfaction there is no objection to reduce the frequency of the injection to eight instead of 4 weeks. This is in line with the former results of our study group, where no effects of a lower injection frequency on visual acuity and central retinal thickness were observed [26].

In daily ophthalmic care the fixed regimen as examined in this study is not routine clinical practice. The treat-and-extent regimen is accepted as the preferred practice, in which, after an initial induction phase, the next treatment interval is extended as long as the patient shows no symptoms of relapse. A lower injection frequency may reduce the burden for patient and doctor, the chances of injection-related side effects, and treatment costs. Hereby, the biggest fear of extending treatment interval is that in the meanwhile the dormant disease will flame up and cause irreversible vision loss. The current challenge is to find the right balance in treating, waiting and adjusting. Another way to reduce burden is to determine whether the initial 4 weeks injection interval used with treat-and-extend could be perhaps 6 or 8 weeks. This current study implicates that there is room to investigate this statement. For an 8 weeks pro re nata, on demand, versus a 4 weeks pro re nata regimen no significant difference was shown [39]. In consideration of these results, low frequency injections (in particular every 8 weeks) of intravitreal bevacizumab should not be withheld from patients with nARMD.

## Strengths and limitations

The every-four-weeks regimen group had the highest drop-out rate. However, it is unlikely that this higher drop-out rate jeopardizes the conclusion, as drop-outs tended to have the same baseline values. The main reasons for treatment discontinuation in all groups were compliance‐related study visit violations. The noncompliance is not only an issue in this study but a problem also in clinical practice [37]. In this study, we see a slightly higher, though not significant, non-adherence rate with the most rigorous treatment schedule, which may be a justification for considering a lower treatment frequency as alternative, as this may increase patient compliance. But where some see frequent visits as a hassle, others will see it as a welcome social benefit. In the end, again, more personalized care might be the answer.

Imbalances were found in the vision-related QoL baseline scores. Principally these differences are a coincidental result of randomization, but as it might have affected the results, the positive effect of the treatment was larger in the eight-week group, we corrected for baseline in the model. In this analysis the interaction between baseline and time confirms the influence of an imbalanced baseline. Apparently, patients with lower baseline scores on average have larger increase in QoL. This could logically be a result of regression to the mean. This same situation occurred in the previous study where the difference of 4 letters on baseline was equalized at follow-up [26]. It is obviously more difficult to improve more if you already have a high QoL.

## Conclusion

Non-inferiority of the 6 and 8 weeks frequencies to 4 weeks was demonstrated on vision-related and general QoL in patients with nARMD. These results are in line with previously published results of these frequency injections. Lower injection frequency may reduce burden, side effects, and treatment costs. In consideration of these results, 6 and in particular 8-week frequency injections of intravitreal bevacizumab could be considered in patients with nARMD.

## Electronic supplementary material

Below is the link to the electronic supplementary material.

(DOCX 32 kb)
