# Supplementary material for: Six and eight weeks injection frequencies of bevacizumab are non-inferior to the current four weeks injection frequency for quality of life in neovascular age-related macular degeneration: a randomized controlled trial
Source: Qual Life Res. 2020 Jul 14;29(12):3305–13. doi: 10.1007/s11136-020-02580-9 (PMC7686180; doi:10.1007/s11136-020-02580-9)
Supplement: Supplementary file 1 — (DOCX 32 kb) [file 11136_2020_2580_MOESM1_ESM.docx]

**Appendix 1. Observed uncorrected changes in the NEI VFQ-39 and SF-36 scores.**

|  | **Every 4 weeks** | | | **Every 6 weeks** | | | **Every 8 weeks** | | | **Differences**  **6-4 weeks^a^** | | | | **Differences**  **8-4 weeks^a^** | | | | **Differences**  **8-6 weeks^a^** | | | | |
| --- | --- | --- | --- | --- | --- | --- | --- | --- | --- | --- | --- | --- | --- | --- | --- | --- | --- | --- | --- | --- | --- | --- |
| **NEI VFQ-39** | Estimate [95% CI] | | | Estimate [95% CI] | | | Estimate [95% CI] | | | Estimate [95% CI] | | | *p*-value | Estimate [95% CI] | | *p*-value | | Estimate [95% CI] | | | *p*-value | |
| Composite score | 1.45 | [-2.56, 5.46] | | 6.52 | [3.14, 9.90] | | 6.49 | [2.07, 10.92] | | 5.07 | [-0.50, 10.63] | | 0.074 | 5.04 | [-0.62, 10.70] | 0.080 | | -0.03 | [-5.35, 5.30] | | 0.992 | |
| Near Activities | 7.71 | [3.23, 12.19] | | 9.98 | [4.78, 15.18] | | 8.31 | [2.09, 14.53] | | 2.26 | [-5.42, 9.95] | | 0.561 | 0.60 | [-7.19, 8.38] | 0.880 | | -1.67 | [-9.03, 5.69] | | 0.655 | |
| Distance Activities | 4.09 | [-1.17, 9.36] | | 5.15 | [1.05, 9.24] | | 8.72 | [2.31, 15.13] | | 1.05 | [-6.44, 8.55] | | 0.782 | 4.63 | [-2.99, 12.24] | 0.232 | | 3.57 | [-3.60, 10.74] | | 0.327 | |
| Role Limitations | -0.69 | [-8.05, 6.66] | | 5.63 | [0.17, 11.08] | | 8.45 | [1.40, 15.51] | | 6.32 | [-2.94, 15.59] | | 0.180 | 9.15 | [-0.27, 18.57] | 0.057 | | 2.82 | [-6.04, 11.69] | | 0.530 | |
| **SF-36** |  |  |  |  |  |  |  |  |  |  |  |  |  |  |  |  |  |  |  |  | |  |
| Physical component | -1.88 | [-4.67, 0.90] | | -0.94 | [-3.24, 1.35] | | -0.35 | [-2.99, 2.29] | | 0.94 | [-2.63, 4.52] | | 0.603 | 1.54 | [-2.11, 5.18] | 0.406 | | 0.59 | [-2.87, 4.06] | | 0.735 | |
| Mental component | 2.24 | [-0.92, 5.39] | | 0.11 | [-2.76, 2.97] | | 3.61 | [0.61, 6.60] | | -2.13 | [-6.32, 2.06] | | 0.316 | 1.37 | [-2.91, 5.64] | 0.528 | | 3.50 | [-0.56, 7.56] | | 0.090 | |

All entries are based on complete cases

**Appendix 2. Observed NEI VFQ-39 and SF-36 scores at both time points.**

|  | **Every 4 weeks** | | | | | | | **Every 6 weeks** | | | | | | **Every 8 weeks** | | | | | |
| --- | --- | --- | --- | --- | --- | --- | --- | --- | --- | --- | --- | --- | --- | --- | --- | --- | --- | --- | --- |
|  | Baseline | | | Follow-up | | | | Baseline | | | Follow-up | | | Baseline | | | Follow-up | | |
| **NEI VFQ-39** | N | mean | sd | | N | mean | sd | N | mean | sd | N | mean | sd | N | mean | sd | N | mean | sd |
| Composite score | 64 | 72.0 | 17.6 | | 45 | 73.9 | 18.0 | 63 | 67.8 | 20.0 | 57 | 74.3 | 17.6 | 64 | 63.1 | 19.4 | 53 | 69.6 | 19.3 |
| Near Activities | 64 | 60.5 | 24.2 | | 45 | 67.8 | 24.8 | 62 | 57.1 | 24.7 | 57 | 65.5 | 24.8 | 64 | 49.4 | 26.7 | 53 | 57.4 | 27.4 |
| Distance Activities | 64 | 67.8 | 23.6 | | 45 | 71.2 | 23.5 | 63 | 64.3 | 25.2 | 57 | 69.5 | 24.3 | 64 | 57.9 | 25.1 | 53 | 65.9 | 25.7 |
| Role Limitations | 64 | 64.2 | 25.9 | | 45 | 64.6 | 27.2 | 63 | 60.2 | 27.2 | 57 | 65.0 | 26.6 | 64 | 52.6 | 25.5 | 53 | 61.3 | 26.6 |
| **SF-36** |  |  |  | |  |  |  |  |  |  |  |  |  |  |  |  |  |  |  |
| Physical component | 58 | 44.8 | 10.9 | | 43 | 44.4 | 10.7 | 61 | 42.1 | 11.1 | 51 | 43.3 | 10.1 | 59 | 42.2 | 9.2 | 48 | 43.0 | 11.0 |
| Mental component | 64 | 72.0 | 17.6 | | 45 | 73.9 | 18.0 | 63 | 51.5 | 11.5 | 57 | 74.3 | 17.6 | 64 | 63.1 | 19.4 | 53 | 69.6 | 19.3 |
